# Oxygen cost of dynamic or isometric exercise relative to recruited muscle mass

**DOI:** 10.1186/1476-5918-5-9

**Published:** 2006-09-11

**Authors:** Christopher P Elder, Edward T Mahoney, Christopher D Black, Jill M Slade, Gary A Dudley

**Affiliations:** 1 Department of Kinesiology, The University of Georgia, Athens, GA, USA; 2 Crawford Research Institute, Shepherd Center, Atlanta, GA, USA

## Abstract

**Background:**

Oxygen cost of different muscle actions may be influenced by different recruitment and rate coding strategies. The purpose of this study was to account for these strategies by comparing the oxygen cost of dynamic and isometric muscle actions relative to the muscle mass recruited via surface electrical stimulation of the knee extensors.

**Methods:**

Comparisons of whole body pulmonary Δ V˙
 MathType@MTEF@5@5@+=feaafiart1ev1aaatCvAUfKttLearuWrP9MDH5MBPbIqV92AaeXatLxBI9gBaebbnrfifHhDYfgasaacH8akY=wiFfYdH8Gipec8Eeeu0xXdbba9frFj0=OqFfea0dXdd9vqai=hGuQ8kuc9pgc9s8qqaq=dirpe0xb9q8qiLsFr0=vr0=vr0dc8meaabaqaciaacaGaaeqabaqabeGadaaakeaacuWGwbGvgaGaaaaa@2DEA@O_2 _were made in seven young healthy adults (1 female) during 3 minutes of dynamic or isometric knee extensions, both induced by surface electrical stimulation. Recruited mass was quantified in T_2 _weighted spin echo magnetic resonance images.

**Results:**

The Δ V˙
 MathType@MTEF@5@5@+=feaafiart1ev1aaatCvAUfKttLearuWrP9MDH5MBPbIqV92AaeXatLxBI9gBaebbnrfifHhDYfgasaacH8akY=wiFfYdH8Gipec8Eeeu0xXdbba9frFj0=OqFfea0dXdd9vqai=hGuQ8kuc9pgc9s8qqaq=dirpe0xb9q8qiLsFr0=vr0=vr0dc8meaabaqaciaacaGaaeqabaqabeGadaaakeaacuWGwbGvgaGaaaaa@2DEA@O_2 _for dynamic muscle actions, 242 ± 128 ml • min^-1 ^(mean ± SD) was greater (p = 0.003) than that for isometric actions, 143 ± 99 ml • min^-1^. Recruited muscle mass was also greater (p = 0.004) for dynamic exercise, 0.716 ± 282 versus 0.483 ± 0.139 kg. The rate of oxygen consumption per unit of recruited muscle (V˙O2RM
 MathType@MTEF@5@5@+=feaafiart1ev1aaatCvAUfKttLearuWrP9MDH5MBPbIqV92AaeXatLxBI9gBaebbnrfifHhDYfgasaacH8akY=wiFfYdH8Gipec8Eeeu0xXdbba9frFj0=OqFfea0dXdd9vqai=hGuQ8kuc9pgc9s8qqaq=dirpe0xb9q8qiLsFr0=vr0=vr0dc8meaabaqaciaacaGaaeqabaqabeGadaaakeaacuqGwbGvgaGaaiabb+eapnaaBaaaleaacqaIYaGmdaahaaadbeqaaiabbkfasjabb2eanbaaaSqabaaaaa@32B0@) was similar in dynamic and isometric exercise (346 ± 162 versus 307 ± 198 ml • kg^-1 ^• min^-1^; p = 0.352), but the V˙O2RM
 MathType@MTEF@5@5@+=feaafiart1ev1aaatCvAUfKttLearuWrP9MDH5MBPbIqV92AaeXatLxBI9gBaebbnrfifHhDYfgasaacH8akY=wiFfYdH8Gipec8Eeeu0xXdbba9frFj0=OqFfea0dXdd9vqai=hGuQ8kuc9pgc9s8qqaq=dirpe0xb9q8qiLsFr0=vr0=vr0dc8meaabaqaciaacaGaaeqabaqabeGadaaakeaacuqGwbGvgaGaaiabb+eapnaaBaaaleaacqaIYaGmdaahaaadbeqaaiabbkfasjabb2eanbaaaSqabaaaaa@32B0@ calculated relative to initial knee extensor torque was significantly greater during dynamic exercise 5.1 ± 1.5 versus 3.6 ± 1.6 ml • kg^-1 ^• Nm^-1 ^• min^-1 ^(p = 0.019).

**Conclusion:**

These results are consistent with the view that oxygen cost of dynamic and  isometric actions is determined by different circumstances of mechanical  interaction between actin and myosin in the sarcomere, and that muscle  recruitment has only a minor role.

## Background

The concept of muscle actions has been introduced to classify the types of length change that skeletal muscle may undergo after it has been activated by the nervous system [1]. The type of length change that occurs is dependent on the balance between the external torque applied to the muscle and the torque generated by the muscle. An action may involve shortening, lengthening, or maintenance of the current muscle length. Shortening and isometric actions share a particular relationship first described based on myothermic measurements by Fenn and thus called the Fenn effect. Fenn showed that extra heat is released when muscle performing an isometric action is allowed to shorten and that the extra heat is approximately proportional to the work done [2,3]. The myothermic measures were later connected with the depletion of phosphocreatine [4] and subsequently associated with the interaction of actin and myosin and the splitting of ATP in the sliding filament theory [5] (see [6] for review). The majority of ATP is supplied systemically through oxidative metabolism of glucose and fatty acids during steady state exercise. Metabolic cost may be measured as the difference between the rate of oxygen consumption at rest and the rate during steady state activity.

In muscle groups, distinct muscle actions may be associated with different recruitment [7] and rate coding strategies by the nervous system [8] in an effort to regulate force output, movement velocity, and metabolism at higher levels than the sarcomere or individual fiber. This may affect energy cost due to the recruitment of large and small motor units composed of specific fiber types, which vary in metabolic capacity and regulation [9].

The investigation of metabolism in whole muscle groups is exemplified by the dynamic knee extensor model of Andersen and Saltin [10]. The dynamic knee extension model has been employed to study muscle blood flow and oxygen consumption and appears well suited for comparisons of systemic oxygen cost differences between different muscle actions. Many investigators recognize the need for precise estimates of muscle mass in expressions of muscle V˙
 MathType@MTEF@5@5@+=feaafiart1ev1aaatCvAUfKttLearuWrP9MDH5MBPbIqV92AaeXatLxBI9gBaebbnrfifHhDYfgasaacH8akY=wiFfYdH8Gipec8Eeeu0xXdbba9frFj0=OqFfea0dXdd9vqai=hGuQ8kuc9pgc9s8qqaq=dirpe0xb9q8qiLsFr0=vr0=vr0dc8meaabaqaciaacaGaaeqabaqabeGadaaakeaacuWGwbGvgaGaaaaa@2DEA@O_2 _. Studies to date have expressed oxygen uptake during dynamic muscle actions relative to the total mass of the knee extensors assessed by anthropometric methods [11-13] or with magnetic resonance imaging (MRI) [14,15]. These studies require the assumption that the entire knee extensor mass may be recruited; which remains a topic of some debate [16]. We sought to extend the previous MRI measures by expressing muscle V˙
 MathType@MTEF@5@5@+=feaafiart1ev1aaatCvAUfKttLearuWrP9MDH5MBPbIqV92AaeXatLxBI9gBaebbnrfifHhDYfgasaacH8akY=wiFfYdH8Gipec8Eeeu0xXdbba9frFj0=OqFfea0dXdd9vqai=hGuQ8kuc9pgc9s8qqaq=dirpe0xb9q8qiLsFr0=vr0=vr0dc8meaabaqaciaacaGaaeqabaqabeGadaaakeaacuWGwbGvgaGaaaaa@2DEA@O_2 _relative to recruited muscle mass. This measure may be termed the recruited mass specific oxygen consumption (V˙O2RM
 MathType@MTEF@5@5@+=feaafiart1ev1aaatCvAUfKttLearuWrP9MDH5MBPbIqV92AaeXatLxBI9gBaebbnrfifHhDYfgasaacH8akY=wiFfYdH8Gipec8Eeeu0xXdbba9frFj0=OqFfea0dXdd9vqai=hGuQ8kuc9pgc9s8qqaq=dirpe0xb9q8qiLsFr0=vr0=vr0dc8meaabaqaciaacaGaaeqabaqabeGadaaakeaacuqGwbGvgaGaaiabb+eapnaaBaaaleaacqaIYaGmdaahaaadbeqaaiabbkfasjabb2eanbaaaSqabaaaaa@32B0@).

Recruited skeletal muscle has been mapped to muscle cross sections [17,18], 3-D volumes [19], and specific pixels [20] in T_2 _weighted MR images based on the increase in T_2 _times exhibited in muscle imaged before and after exercise. It has been observed that T_2 _increases are directly related to EMG [21], exercise intensity [22,23], duration [23] and aerobic capacity [24]. Increases in T_2 _have also been used to distinguish between muscles that perform lengthening or shortening actions [25]. Expressing the V˙O2RM
 MathType@MTEF@5@5@+=feaafiart1ev1aaatCvAUfKttLearuWrP9MDH5MBPbIqV92AaeXatLxBI9gBaebbnrfifHhDYfgasaacH8akY=wiFfYdH8Gipec8Eeeu0xXdbba9frFj0=OqFfea0dXdd9vqai=hGuQ8kuc9pgc9s8qqaq=dirpe0xb9q8qiLsFr0=vr0=vr0dc8meaabaqaciaacaGaaeqabaqabeGadaaakeaacuqGwbGvgaGaaiabb+eapnaaBaaaleaacqaIYaGmdaahaaadbeqaaiabbkfasjabb2eanbaaaSqabaaaaa@32B0@ requires mapping muscle activity to specific pixels. Adams et al. [20] introduced a 1 standard deviation threshold method to investigate the distribution of electrical stimulation throughout individual muscle cross-sections. The threshold mapping technique has been applied after voluntary exercise [17], but Prior et al. [26] provided evidence questioning the validity of voluntary applications and supporting the validity of mapping active muscle to specific pixels after electrical stimulation.

Surface neuromuscular electrical stimulation (ES) is thought to activate skeletal muscle through the axonal branches of motor neurons, bypassing the central nervous system [27]. Synchronous motor unit activation by ES appears eliminate additional recruitment [28] and changes in firing rate [29] that are observed during voluntary contractions. Changes in motor unit behavior have been suggested as a mechanism for the increasing V˙
 MathType@MTEF@5@5@+=feaafiart1ev1aaatCvAUfKttLearuWrP9MDH5MBPbIqV92AaeXatLxBI9gBaebbnrfifHhDYfgasaacH8akY=wiFfYdH8Gipec8Eeeu0xXdbba9frFj0=OqFfea0dXdd9vqai=hGuQ8kuc9pgc9s8qqaq=dirpe0xb9q8qiLsFr0=vr0=vr0dc8meaabaqaciaacaGaaeqabaqabeGadaaakeaacuWGwbGvgaGaaaaa@2DEA@O_2 _[30] and metabolic heat production [31] observed during prolonged voluntary intermittent isometric contractions. Expressing V˙O2RM
 MathType@MTEF@5@5@+=feaafiart1ev1aaatCvAUfKttLearuWrP9MDH5MBPbIqV92AaeXatLxBI9gBaebbnrfifHhDYfgasaacH8akY=wiFfYdH8Gipec8Eeeu0xXdbba9frFj0=OqFfea0dXdd9vqai=hGuQ8kuc9pgc9s8qqaq=dirpe0xb9q8qiLsFr0=vr0=vr0dc8meaabaqaciaacaGaaeqabaqabeGadaaakeaacuqGwbGvgaGaaiabb+eapnaaBaaaleaacqaIYaGmdaahaaadbeqaaiabbkfasjabb2eanbaaaSqabaaaaa@32B0@ by quantifying the recruited mass in T_2 _MRI after ES exercise may allow a unique comparison of the oxygen cost between dynamic and isometric muscle actions in the knee extensor muscle group in vivo. The hypothesis was that dynamic muscle actions would result in greater V˙O2RM
 MathType@MTEF@5@5@+=feaafiart1ev1aaatCvAUfKttLearuWrP9MDH5MBPbIqV92AaeXatLxBI9gBaebbnrfifHhDYfgasaacH8akY=wiFfYdH8Gipec8Eeeu0xXdbba9frFj0=OqFfea0dXdd9vqai=hGuQ8kuc9pgc9s8qqaq=dirpe0xb9q8qiLsFr0=vr0=vr0dc8meaabaqaciaacaGaaeqabaqabeGadaaakeaacuqGwbGvgaGaaiabb+eapnaaBaaaleaacqaIYaGmdaahaaadbeqaaiabbkfasjabb2eanbaaaSqabaaaaa@32B0@ compared to isometric actions when each was induced by similar protocols of ES.

## Methods

In this study, each subject completed a submaximal bout of two-legged dynamic or isometric knee extensions using the quadriceps femoris muscle group activated with ES. Pulmonary V˙
 MathType@MTEF@5@5@+=feaafiart1ev1aaatCvAUfKttLearuWrP9MDH5MBPbIqV92AaeXatLxBI9gBaebbnrfifHhDYfgasaacH8akY=wiFfYdH8Gipec8Eeeu0xXdbba9frFj0=OqFfea0dXdd9vqai=hGuQ8kuc9pgc9s8qqaq=dirpe0xb9q8qiLsFr0=vr0=vr0dc8meaabaqaciaacaGaaeqabaqabeGadaaakeaacuWGwbGvgaGaaaaa@2DEA@O_2 _was measured at rest and during each type of exercise. On a separate day, the dynamic and isometric exercise bouts were repeated and T_2 _weighted MR images were acquired before and immediately following each exercise bout.

### Subjects

Six males and one female age 28 ± 3 yr (mean ± (SD)), height of 176 ± 7 cm and a mass of 81 ± 12 kg, volunteered to participate in this study. All volunteers provided written informed consent. The methods were approved by The Institutional Review Boards of The University of Georgia and Shepherd Center.

### Dynamic exercise

Two-legged dynamic knee extensions were performed on a modified, pushing style Krogh ergometer [10] with no load. ES electrodes (7 × 10 cm, Uni-Patch Wabasha, MN.) were placed on the skin over the distal vastus medialis and the proximal vastus lateralis. ES was gated by a sensor attached to the sprocket of the ergometer and only resulted in muscle contraction when the sprocket passed through the shortening range of motion for knee extension; lengthening movement was passive. Muscle actions were elicited with 450 μs biphasic square wave pulses at a frequency of 30 Hz and amplitude sufficient to allow the subject to maintain 30 revolutions per minute on the ergometer. The flywheel was already spinning at approximately 30 rpm at the beginning of the exercise to eliminate the metabolic cost of overcoming inertia. ES current evoked dynamic contractions in for 3 minutes at 1:2 duty cycle with the shortening component accounting for an average of 0.96 ± 0.1 s per contraction cycle. ES amplitude was 51 ± 13 mAmps for the right and 50 ± 14 mAmps for the left thigh. Muscle actions were elicited at a rate of 32 ± 3 contractions per minute. The shortening component of the dynamic action was accomplished at an average of 74 ± 7 degrees per second and the average power output during dynamic exercise was 4.8 ± 0.5 watts. Dynamic torque was estimated from isometric torque at 70° of flexion using the expressions of the torque velocity relationship first described by Hill et al. [32] and the dynamic constants derived by Dudley et al. [33] for electrically stimulated knee extensions.

### Isometric exercise

Two-legged isometric actions of the knee extensors were performed on a custom-built chair with the hip and knee secured at ~70° of flexion. The leg was firmly secured to a rigid lever arm with an inelastic strap to ensure that the knee extensors could only perform isometric contractions. The moment arm was established by placing a load cell (model 2000A, Rice Lake Weighing Systems, Rice Lake, WI) parallel to the line of pull and perpendicular to the lever arm. Torque was recorded from the load cell by using a MacLab analog-to-digital converter (model ML 400, ADInstruments, Milford, MA) sampling at 100 Hz and interfaced with a portable Macintosh computer (Apple Computer, Cupertino, CA). Isometric actions were elicited by ES matched with that of dynamic exercise including 450 μs biphasic pulses, a frequency of 30 Hz and amplitude of 51 ± 13 mAmps for the right and 50 ± 14 mAmps for the left thigh. ES current evoked isometric actions for 3 minutes with a 1:2 duty cycle resulting in a rate of 30 contractions per minute. ES was therefore essentially identical to that used during dynamic action knee extensions in order to activate muscle in the same manner during both types of exercise.

### Oxygen uptake

Oxygen uptake was measured via expired gasses on a Vista mini-CPX system (Vacumed Inc., Ventura, CA) during three minutes of rest and three minutes of either dynamic or isometric exercise. Pulmonary oxygen uptake was collected breath by breath and averaged every 10 seconds. Resting V˙
 MathType@MTEF@5@5@+=feaafiart1ev1aaatCvAUfKttLearuWrP9MDH5MBPbIqV92AaeXatLxBI9gBaebbnrfifHhDYfgasaacH8akY=wiFfYdH8Gipec8Eeeu0xXdbba9frFj0=OqFfea0dXdd9vqai=hGuQ8kuc9pgc9s8qqaq=dirpe0xb9q8qiLsFr0=vr0=vr0dc8meaabaqaciaacaGaaeqabaqabeGadaaakeaacuWGwbGvgaGaaaaa@2DEA@O_2 _was measured as 3-minute average of the 10-second intervals collected prior to exercise. The average resting V˙
 MathType@MTEF@5@5@+=feaafiart1ev1aaatCvAUfKttLearuWrP9MDH5MBPbIqV92AaeXatLxBI9gBaebbnrfifHhDYfgasaacH8akY=wiFfYdH8Gipec8Eeeu0xXdbba9frFj0=OqFfea0dXdd9vqai=hGuQ8kuc9pgc9s8qqaq=dirpe0xb9q8qiLsFr0=vr0=vr0dc8meaabaqaciaacaGaaeqabaqabeGadaaakeaacuWGwbGvgaGaaaaa@2DEA@O_2 _was then subtracted from each 10 second interval during exercise to yield the Δ V˙
 MathType@MTEF@5@5@+=feaafiart1ev1aaatCvAUfKttLearuWrP9MDH5MBPbIqV92AaeXatLxBI9gBaebbnrfifHhDYfgasaacH8akY=wiFfYdH8Gipec8Eeeu0xXdbba9frFj0=OqFfea0dXdd9vqai=hGuQ8kuc9pgc9s8qqaq=dirpe0xb9q8qiLsFr0=vr0=vr0dc8meaabaqaciaacaGaaeqabaqabeGadaaakeaacuWGwbGvgaGaaaaa@2DEA@O_2_. Steady state oxygen demand was estimated from the power output of each subject during dynamic exercise by interpolating data to an exponential fit (R^2 ^= 0.90) of the relationship between electrically evoked dynamic knee extension power output and pulmonary V˙
 MathType@MTEF@5@5@+=feaafiart1ev1aaatCvAUfKttLearuWrP9MDH5MBPbIqV92AaeXatLxBI9gBaebbnrfifHhDYfgasaacH8akY=wiFfYdH8Gipec8Eeeu0xXdbba9frFj0=OqFfea0dXdd9vqai=hGuQ8kuc9pgc9s8qqaq=dirpe0xb9q8qiLsFr0=vr0=vr0dc8meaabaqaciaacaGaaeqabaqabeGadaaakeaacuWGwbGvgaGaaaaa@2DEA@O_2 _constructed from the data of Kim et al. [34]. The exponential fit was corrected for the assumed metabolic differences between stimulation frequencies used by Kim (50 Hz) and that of the present study (30 Hz) using the force frequency relationship found in Bellemare et al. [35]. After interpolation, oxygen demands were corrected for each subject's average resting V˙
 MathType@MTEF@5@5@+=feaafiart1ev1aaatCvAUfKttLearuWrP9MDH5MBPbIqV92AaeXatLxBI9gBaebbnrfifHhDYfgasaacH8akY=wiFfYdH8Gipec8Eeeu0xXdbba9frFj0=OqFfea0dXdd9vqai=hGuQ8kuc9pgc9s8qqaq=dirpe0xb9q8qiLsFr0=vr0=vr0dc8meaabaqaciaacaGaaeqabaqabeGadaaakeaacuWGwbGvgaGaaaaa@2DEA@O_2_. Each 10-second interval Δ V˙
 MathType@MTEF@5@5@+=feaafiart1ev1aaatCvAUfKttLearuWrP9MDH5MBPbIqV92AaeXatLxBI9gBaebbnrfifHhDYfgasaacH8akY=wiFfYdH8Gipec8Eeeu0xXdbba9frFj0=OqFfea0dXdd9vqai=hGuQ8kuc9pgc9s8qqaq=dirpe0xb9q8qiLsFr0=vr0=vr0dc8meaabaqaciaacaGaaeqabaqabeGadaaakeaacuWGwbGvgaGaaaaa@2DEA@O_2 _was then compared to the corresponding estimated oxygen demand and the comparisons analyzed using repeated measures ANOVA. Dynamic Δ V˙
 MathType@MTEF@5@5@+=feaafiart1ev1aaatCvAUfKttLearuWrP9MDH5MBPbIqV92AaeXatLxBI9gBaebbnrfifHhDYfgasaacH8akY=wiFfYdH8Gipec8Eeeu0xXdbba9frFj0=OqFfea0dXdd9vqai=hGuQ8kuc9pgc9s8qqaq=dirpe0xb9q8qiLsFr0=vr0=vr0dc8meaabaqaciaacaGaaeqabaqabeGadaaakeaacuWGwbGvgaGaaaaa@2DEA@O_2 _was not statistically different from dynamic oxygen demand for any interval after 20 seconds of exercise up to the final interval. The oxygen uptake component (numerator) of the V˙O2RM
 MathType@MTEF@5@5@+=feaafiart1ev1aaatCvAUfKttLearuWrP9MDH5MBPbIqV92AaeXatLxBI9gBaebbnrfifHhDYfgasaacH8akY=wiFfYdH8Gipec8Eeeu0xXdbba9frFj0=OqFfea0dXdd9vqai=hGuQ8kuc9pgc9s8qqaq=dirpe0xb9q8qiLsFr0=vr0=vr0dc8meaabaqaciaacaGaaeqabaqabeGadaaakeaacuqGwbGvgaGaaiabb+eapnaaBaaaleaacqaIYaGmdaahaaadbeqaaiabbkfasjabb2eanbaaaSqabaaaaa@32B0@ expression is derived from the 60-second average of the 10-second intervals comprising minute three of exercise for both dynamic and isometric exercise.

### MR imaging

Standard T_2 _weighted spin-echo images of the thighs were collected using a 1.5 Tesla super-conducting magnet (Signa, General Electric, Milwaukee, WI). Transaxial images (TR/TE = 2000/30, 60) 1.0 cm thick, spaced 1.0 cm apart were acquired in a 256 × 256 matrix with one excitation and a 40 cm field of view using a whole body coil. MR images were analyzed for total muscle mass and recruited muscle mass as described previously [18] using NIH Image 1.62 [36]. After spatial calibration (40 cm/256 pixels = 0.024 cm^2^/pixel) and manual tracing of the region of interest, the cross sectional area (CSA) of pixels considered recruited was determined for each set of pre and post exercise images. Pixels representing resting skeletal muscle in the pre exercise images were defined as those with a T_2 _between 20 and 35 ms. Recruited muscle was defined by pixels in the post exercise images with a T_2 _> mean + 1 SD of the muscle T_2 _in the pre exercise images [18,21]. Pixels with a T_2 _above 55 were also excluded in the post-ES images to correct for areas of lipid. CSA values were summed over successive slices beginning with the first slice not containing the proximal head of the femur and continuing distally until the slice just before the top of the patella. Recruited muscle volume was calculated by multiplying contracting CSA by the slice thickness and spacing. Volume in cm^3 ^was then converted to grams assuming a skeletal muscle density of 1.06 grams • cm^-3 ^[37]. To avoid overestimation of recruited skeletal muscle, there was at least forty-five minute rest between exercise bouts, which was adequate for complete decay of the T_2 _signal [24]. Pre- and post-ES images were matched to ensure that the same muscle area was considered for dynamic and isometric exercise.

### Statistics

The results are expressed as mean ± SD. All statistical tests were performed on SPSS version 13.0. Data from dynamic and isometric knee extensions were compared using a dependent t-test. The significance level was α = 0.05.

## Results

Subjects oxygen consumption expressed as the average pulmonary Δ V˙
 MathType@MTEF@5@5@+=feaafiart1ev1aaatCvAUfKttLearuWrP9MDH5MBPbIqV92AaeXatLxBI9gBaebbnrfifHhDYfgasaacH8akY=wiFfYdH8Gipec8Eeeu0xXdbba9frFj0=OqFfea0dXdd9vqai=hGuQ8kuc9pgc9s8qqaq=dirpe0xb9q8qiLsFr0=vr0=vr0dc8meaabaqaciaacaGaaeqabaqabeGadaaakeaacuWGwbGvgaGaaaaa@2DEA@O_2 _between rest and the third minute of exercise reached a value 242 ± 128 ml • min^-1 ^during dynamic exercise which was greater than the value for isometric exercise of 143 ± 99 ml • min^-1 ^(p = 0.003) (Table 1). Work of the heart contributed to the Δ V˙
 MathType@MTEF@5@5@+=feaafiart1ev1aaatCvAUfKttLearuWrP9MDH5MBPbIqV92AaeXatLxBI9gBaebbnrfifHhDYfgasaacH8akY=wiFfYdH8Gipec8Eeeu0xXdbba9frFj0=OqFfea0dXdd9vqai=hGuQ8kuc9pgc9s8qqaq=dirpe0xb9q8qiLsFr0=vr0=vr0dc8meaabaqaciaacaGaaeqabaqabeGadaaakeaacuWGwbGvgaGaaaaa@2DEA@O_2_, but was likely similar between exercise modes as reflected by similar (p = 0.113) increases in heart rate of 16 ± 12 for isometric and 22 ± 14 for dynamic exercise.

**Table 1:** Physiological responses to dynamic and isometric exercise

	**Dynamic**	**Isometric**
Δ VO_2 _(ml•min^-1^)	242 ± 128	143 ± 99*
Δ Recruited muscle (kg)	0.716 ± 0.147	0.483 ± 0.139*

Change in V˙
 MathType@MTEF@5@5@+=feaafiart1ev1aaatCvAUfKttLearuWrP9MDH5MBPbIqV92AaeXatLxBI9gBaebbnrfifHhDYfgasaacH8akY=wiFfYdH8Gipec8Eeeu0xXdbba9frFj0=OqFfea0dXdd9vqai=hGuQ8kuc9pgc9s8qqaq=dirpe0xb9q8qiLsFr0=vr0=vr0dc8meaabaqaciaacaGaaeqabaqabeGadaaakeaacuWGwbGvgaGaaaaa@2DEA@O_2 _and recruited muscle mass from rest for dynamic and isometric exercise. Values are presented as mean ± SD, *p < 0.05

**Figure 1:**
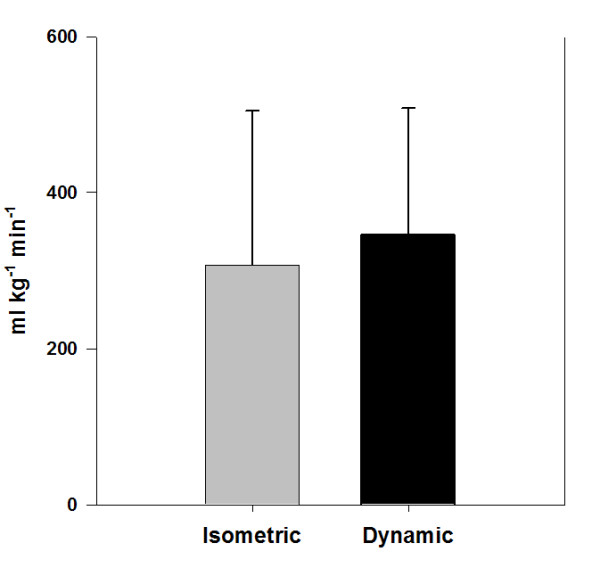
**Oxygen consumption expressed relative to recruited muscle mass**. Dynamic is not significantly different from isometric. Values are presented as mean + SD; p = 0.352.

Total muscle mass of the right and the left quadriceps femoris was 4.16 ± 0.86 kg. The muscle mass recruited during exercise was greater for dynamic than for isometric actions, 0.716 ± 0.147 versus 0.483 ± 0.139 kg (p = 0.004) (Table 1). Calculating the ratio of oxygen consumption to recruited mass resulted in a V˙O2RM
 MathType@MTEF@5@5@+=feaafiart1ev1aaatCvAUfKttLearuWrP9MDH5MBPbIqV92AaeXatLxBI9gBaebbnrfifHhDYfgasaacH8akY=wiFfYdH8Gipec8Eeeu0xXdbba9frFj0=OqFfea0dXdd9vqai=hGuQ8kuc9pgc9s8qqaq=dirpe0xb9q8qiLsFr0=vr0=vr0dc8meaabaqaciaacaGaaeqabaqabeGadaaakeaacuqGwbGvgaGaaiabb+eapnaaBaaaleaacqaIYaGmdaahaaadbeqaaiabbkfasjabb2eanbaaaSqabaaaaa@32B0@ for dynamic exercise of 346 ± 162 ml • kg^-1 ^• min^-1 ^and was not statistically different from the value of 307 ± 198 ml • kg^-1 ^• min^-1 ^calculated for isometric exercise (p = 0.352) (Figure 1).

**Figure 2:**
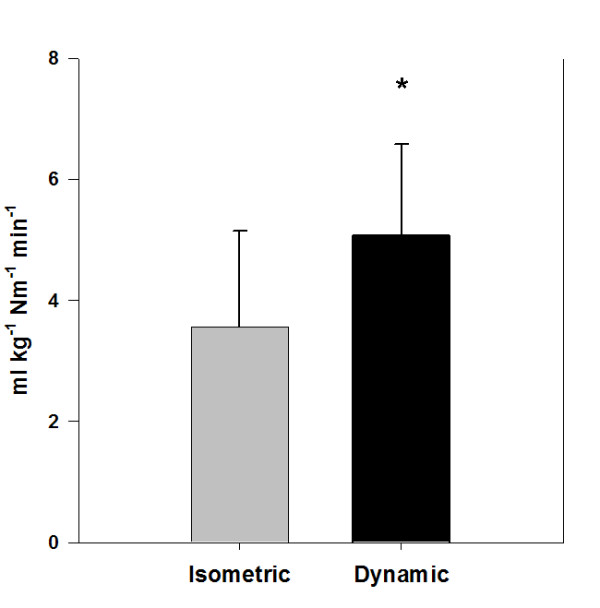
**Oxygen consumption expressed relative to recruited muscle mass and accounting for differences in torque between muscle actions**. Dynamic is significantly different from isometric. Values are presented as mean + SD; p = 0.019.

Initial isometric torque of 84 ± 30 Nm was greater than the estimated initial dynamic torque of 67 ± 22 Nm at 70° of flexion (p = 0.001); therefore V˙O2RM
 MathType@MTEF@5@5@+=feaafiart1ev1aaatCvAUfKttLearuWrP9MDH5MBPbIqV92AaeXatLxBI9gBaebbnrfifHhDYfgasaacH8akY=wiFfYdH8Gipec8Eeeu0xXdbba9frFj0=OqFfea0dXdd9vqai=hGuQ8kuc9pgc9s8qqaq=dirpe0xb9q8qiLsFr0=vr0=vr0dc8meaabaqaciaacaGaaeqabaqabeGadaaakeaacuqGwbGvgaGaaiabb+eapnaaBaaaleaacqaIYaGmdaahaaadbeqaaiabbkfasjabb2eanbaaaSqabaaaaa@32B0@ calculated relative to initial knee extensor torque was significantly greater during dynamic exercise 5.1 ± 1.5 ml • kg^-1 ^• Nm^-1 ^• min^-1 ^versus 3.6 ± 1.6 ml kg^-1 ^• Nm^-1 ^• min^-1 ^(p = 0.019) (Figure 2). Oxygen consumption expressed relative to initial torque only was 4.4 ± 1.4 ml • Nm^-1 ^• min^-1 ^for dynamic actions and 2.2 ± 1.1 ml • Nm • min^-1 ^for isometric actions (p = 0.007).

## Discussion

The purpose of this study was to make a unique comparison of the oxygen cost between dynamic and isometric muscle actions by quantifying the recruited mass in the knee extensor muscle group during surface electrical stimulation exercise in vivo. We hypothesized that dynamic exercise would exhibit a greater energy even after accounting for recruited muscle mass. The data suggest that the oxygen cost of exercise per unit mass of recruited muscle is approximately equal between dynamic and isometric exercise of the knee extensors under conditions of surface electrical stimulation. Expressing the oxygen cost per unit recruited mass relative to initial torque does reveal differences between muscle actions but does not completely eliminate the effects of recruitment as evidenced by even larger differences between actions expressed per initial torque only.

Pulmonary V˙
 MathType@MTEF@5@5@+=feaafiart1ev1aaatCvAUfKttLearuWrP9MDH5MBPbIqV92AaeXatLxBI9gBaebbnrfifHhDYfgasaacH8akY=wiFfYdH8Gipec8Eeeu0xXdbba9frFj0=OqFfea0dXdd9vqai=hGuQ8kuc9pgc9s8qqaq=dirpe0xb9q8qiLsFr0=vr0=vr0dc8meaabaqaciaacaGaaeqabaqabeGadaaakeaacuWGwbGvgaGaaaaa@2DEA@O_2 _is not limited to the oxygen uptake of the active muscle but also includes the oxygen consumed in the heart, and other tissues. Pulmonary oxygen uptake depends on the heart rate, stroke volume, and a-v O_2 _difference. Skeletal and heart muscle receive 90 to 95 percent of the increase in cardiac output at the onset of exercise; in addition, oxygen extraction does not increase in inactive tissues to the same extent as it does in muscle during exercise [38]. We conclude that heart and skeletal muscle account for almost all of the Δ V˙
 MathType@MTEF@5@5@+=feaafiart1ev1aaatCvAUfKttLearuWrP9MDH5MBPbIqV92AaeXatLxBI9gBaebbnrfifHhDYfgasaacH8akY=wiFfYdH8Gipec8Eeeu0xXdbba9frFj0=OqFfea0dXdd9vqai=hGuQ8kuc9pgc9s8qqaq=dirpe0xb9q8qiLsFr0=vr0=vr0dc8meaabaqaciaacaGaaeqabaqabeGadaaakeaacuWGwbGvgaGaaaaa@2DEA@O_2 _measured in the present study. Heart V˙
 MathType@MTEF@5@5@+=feaafiart1ev1aaatCvAUfKttLearuWrP9MDH5MBPbIqV92AaeXatLxBI9gBaebbnrfifHhDYfgasaacH8akY=wiFfYdH8Gipec8Eeeu0xXdbba9frFj0=OqFfea0dXdd9vqai=hGuQ8kuc9pgc9s8qqaq=dirpe0xb9q8qiLsFr0=vr0=vr0dc8meaabaqaciaacaGaaeqabaqabeGadaaakeaacuWGwbGvgaGaaaaa@2DEA@O_2 _was likely similar between exercise types as evidenced by similar increases in heart rate.

Our purpose in the present investigation was to confine muscle oxygen consumption to the knee extensor muscle group in both dynamic and isometric exercise. The dynamic knee extension device was devised by Andersen and Saltin [10] as a model for isolated muscle in vivo. Richardson et al. [39] measured the change in T_2 _in thigh muscle groups after voluntary dynamic knee extension and concluded that recruitment is confined to the quadriceps femoris muscle group. In the present study, electrical stimulation controlled muscle recruitment directly to the knee extensors. Visual inspection of contrast patterns in post exercise images confirmed that recruitment was confined to the m. quadriceps femoris.

The electrical stimulation protocols (frequency, amplitude, and pulse duration) were identical between isometric and dynamic exercise. The absolute mass of muscle recruited was much larger for dynamic exercise. It is well known that muscle architecture changes during muscle actions, specifically pennation angle and fascicle length [40-42]. There are larger alterations in fascicle length and pennation angle during dynamic than isometric actions which significantly alter the 3 dimensional shape of the muscle and could bring more nerve fibers branches into the field of the electrical stimulation during shortening than during isometric actions.

A possible limitation to the interpretation of these results is the difficulty in controlling contributions of stabilizing muscles of the hips, torso, and arms. Similarities in body position, posture, and restraints to extraneous movement for isometric and dynamic exercise support the interpretation of comparisons between muscle actions; the absolute magnitude of the oxygen cost must be interpreted with caution. Nonetheless, it is interesting to note the similarity between our measures of dynamic oxygen consumption (346 ml • kg ^-1 ^• min^-1^) and the estimates of 330 ml • kg ^-1 ^• min^-1 ^• made in human knee extensors during maximal voluntary exercise by Magnusson et al. [43] and 377 ml • kg ^-1 ^• min^-1 ^estimated in horses by Armstrong et al. [44].

There are also limitations to the interpretation of the present data expressed per unit of initial torque. The absolute measures should again be interpreted with caution. We were unable to retrieve torque data in all subjects during the final minute of exercise due to technical limitations; therefore, we cannot report the V˙O2RM
 MathType@MTEF@5@5@+=feaafiart1ev1aaatCvAUfKttLearuWrP9MDH5MBPbIqV92AaeXatLxBI9gBaebbnrfifHhDYfgasaacH8akY=wiFfYdH8Gipec8Eeeu0xXdbba9frFj0=OqFfea0dXdd9vqai=hGuQ8kuc9pgc9s8qqaq=dirpe0xb9q8qiLsFr0=vr0=vr0dc8meaabaqaciaacaGaaeqabaqabeGadaaakeaacuqGwbGvgaGaaiabb+eapnaaBaaaleaacqaIYaGmdaahaaadbeqaaiabbkfasjabb2eanbaaaSqabaaaaa@32B0@ relative to torque during the third minute of exercise. In a study conducted by Bickel et al. [45] isometric force during intermittent contractions evoked by electrical stimulation reached ~45% of its initial value after 90 contractions at 16 Hz and a duty cycle similar to the current study. Retest of one subject from current study at a later time resulted in an approximately exponential decrease in isometric force to 40% of its initial value after 35 contractions. The magnitude of force remained stable from contraction 35 to 90, indicating a steady state of force production was reached after 70 seconds of exercise. Time courses with similar characteristics are found in other studies of electrically stimulated fatigue [45-47]. If this time course and magnitude of force loss can be generalized to other subjects in the present study, it was not associated with a decrease in oxygen consumption. In any case, fatigue during dynamic exercise would almost certainly have been greater than isometric under the same stimulation conditions [48], serving only to increase the oxygen cost difference between exercise modes.

Other investigators have found similar results when comparing the metabolic cost of isometric and shortening actions. Ryschon et al. [49] estimated the maximal ATP synthesis rate with ^31^P NMR during dorsiflexion exercise in humans to be almost double for shortening versus isometric muscle actions. Beltman et al. [50] showed that the ratio of the change in high energy phosphates to the force time integral of dynamic, muscle actions was twice as high as the same ratio for isometric actions using maximal electrical stimulation of the rat medial gastrocnemius.

In this study, we hypothesized that differences in oxygen cost between muscle actions may be affected by different recruitment strategies. Expressing oxygen consumption relative to muscle recruited with electrical stimulation eliminated the difference in energy cost between isometric and dynamic modes, suggesting that recruitment does help to determine cost in vivo. However, differences in oxygen cost were found after accounting for the decrease in torque in the recruited muscle associated with shortening velocity. Excitation via electrical stimulation results in random recruitment that is spatially fixed and temporally synchronous (see ref [28] for review). Repeated tests in the same subjects give the probabilistic assurance of equal distribution of recruited fiber types in the populations compared, thus fiber type differences in ATPase rate due to recruitment of differing fiber populations could not explain the differences in energy cost.

There is a specific amount of energy release associated with an isometric action which has been termed the activation heat based on myothermic measures (see reference [6]). Fenn showed that extra heat is released when muscle performing an isometric action is allowed to shorten and the extra heat is proportional to the work done [2,3]. The extra heat was later associated with a greater breakdown of phosphocreatine (see reference [6]). On a molecular level, shortening involves an increase in the rate of crossbridge cycling as myosin moves along the actin filament [5]. Cycling involves many instances of ATP splitting to accomplish the power stroke of force generation and myosin detachment from actin. In isometric actions, some shortening does occur in vivo [40], but the cycling rate is greatly reduced along with ATP consumption. Shortening velocity will also play a role; the rate of energy release becomes greater as velocity of shortening increases [32]. Expression of Δ V˙
 MathType@MTEF@5@5@+=feaafiart1ev1aaatCvAUfKttLearuWrP9MDH5MBPbIqV92AaeXatLxBI9gBaebbnrfifHhDYfgasaacH8akY=wiFfYdH8Gipec8Eeeu0xXdbba9frFj0=OqFfea0dXdd9vqai=hGuQ8kuc9pgc9s8qqaq=dirpe0xb9q8qiLsFr0=vr0=vr0dc8meaabaqaciaacaGaaeqabaqabeGadaaakeaacuWGwbGvgaGaaaaa@2DEA@O_2 _relative to torque production in the recruited muscle expresses the dependence of energy cost on the load and the distance of shortening. These relationships are due to inherent mechanical differences in crossbridge interaction during different muscle actions. If the same total amount of time is allowed for force production in shortening and isometric actions, shortening involves a greater proportion of time for crossbridge cycling and ATP splitting; isometric actions involve a greater proportion of time in which actin and myosin are bound in and not splitting ATP. The systemic result of these mechanical differences between actions is a greater metabolic or oxygen cost for dynamic exercise.

## Conclusion

Differences in oxygen cost were largely dependent on torque generated by  the recruited muscle and not on the mass of muscle recruited via electrical  stimulation at constant frequency. These results are consistent with the  view that oxygen cost of dynamic and isometric actions is determined by  different circumstances of mechanical interaction between actin and myosin  in the sarcomere, and that muscle recruitment has only a minor role. Further  studies of this phenomenon should focus on aspects of fatigue and torque  output.

## Abbreviations

Electrical stimulation ES

Magnetic resonance imaging MRI

Respiratory whole body oxygen consumption rate V˙
 MathType@MTEF@5@5@+=feaafiart1ev1aaatCvAUfKttLearuWrP9MDH5MBPbIqV92AaeXatLxBI9gBaebbnrfifHhDYfgasaacH8akY=wiFfYdH8Gipec8Eeeu0xXdbba9frFj0=OqFfea0dXdd9vqai=hGuQ8kuc9pgc9s8qqaq=dirpe0xb9q8qiLsFr0=vr0=vr0dc8meaabaqaciaacaGaaeqabaqabeGadaaakeaacuWGwbGvgaGaaaaa@2DEA@O_2_

Oxygen consumption of the recruited muscle mass V˙O2RM
 MathType@MTEF@5@5@+=feaafiart1ev1aaatCvAUfKttLearuWrP9MDH5MBPbIqV92AaeXatLxBI9gBaebbnrfifHhDYfgasaacH8akY=wiFfYdH8Gipec8Eeeu0xXdbba9frFj0=OqFfea0dXdd9vqai=hGuQ8kuc9pgc9s8qqaq=dirpe0xb9q8qiLsFr0=vr0=vr0dc8meaabaqaciaacaGaaeqabaqabeGadaaakeaacuqGwbGvgaGaaiabb+eapnaaBaaaleaacqaIYaGmdaahaaadbeqaaiabbkfasjabb2eanbaaaSqabaaaaa@32B0@

Transverse relaxation time T_2_

Adenosine triphosphate ATP

Phosphorous nuclear magnetic resonance ^31^P NMR

## Authors' contributions

CPE conceived of the study, participated in and managed all aspects of its design, coordination, data collection, and analysis, and drafted the manuscript. ETM participated in the study design, coordination, and data collection and assisted with the drafting of the manuscript. CDB assisted with data collection, interpretation and the drafting of the manuscript. JMS assisted with data collection and statistical analysis. GAD directed the laboratory and participated in all aspects of the study design, coordination, data analysis, and interpretation. All authors read and approved the final manuscript.
